# Complementary effects of HDAC inhibitor 4-PB on gap junction communication and cellular export mechanisms support restoration of chemosensitivity of PDAC cells

**DOI:** 10.1038/sj.bjc.6603511

**Published:** 2006-12-12

**Authors:** O Ammerpohl, A Trauzold, B Schniewind, U Griep, C Pilarsky, R Grutzmann, H-D Saeger, O Janssen, B Sipos, G Kloppel, H Kalthoff

**Affiliations:** 1 Section Molecular Oncology, Clinic for General Surgery and Thoracic Surgery, University Hospital Schleswig-Holstein Campus Kiel, Arnold-Heller Strasse 7, Kiel 24105, Germany; 2 Department of Visceral, Thoracic and Vascular Surgery, University Hospital Carl Gustav Carus, Technical University of Dresden, Dresden 01307, Germany; 3 Institute of Immunology, University Hospital Schleswig-Holstein Campus Kiel, Kiel 24105, Germany; 4 Institute of Pathology, University Hospital Schleswig-Holstein Campus Kiel, Kiel 24105, Germany

**Keywords:** HDAC inhibitor, 4-phenylbutyrate, gemcitabine, pancreatic carcinoma

## Abstract

Pancreatic ductal adenocarcinoma (PDAC) is a fatal disease and one of the cancer entities with the lowest life expectancy. Beside surgical therapy, no effective therapeutic options are available yet. Here, we show that 4-phenylbutyrate (4-PB), a known and well-tolerable inhibitor of histone deacetylases (HDAC), induces up to 70% apoptosis in all cell lines tested (Panc 1, T4M-4, COLO 357, BxPc3). In contrast, it leads to cell cycle arrest in only half of the cell lines tested. This drug increases gap junction communication between adjacent T3M-4 cells in a concentration-dependent manner and efficiently inhibits cellular export mechanisms in Panc 1, T4M-4, COLO 357 and BxPc3 cells. Consequently, in combination with gemcitabine 4-PB shows an overadditive effect on induction of apoptosis in BxPc3 and T3M-4 cells (up to 4.5-fold compared to single drug treatment) with accompanied activation of Caspase 8, BH3 interacting domain death agonist (Bid) and poly (ADP-ribose) polymerase family, member 1 (PARP) cleavage. Although the inhibition of the mitogen-activated protein kinase-pathway has no influence on fulminant induction of apoptosis, the inhibition of the JNK-pathway by SP600125 completely abolishes the overadditive effect induced by the combined application of both drugs, firstly reported by this study.

Patients with pancreatic ductal adenocarcinoma (PDAC) still suffer from a very poor prognosis. Pancreatic ductal adenocarcinoma is ranked fourth among the cancer-related death in the US, responsible for approximately 31 000 deaths per year and the overall 5-year survival rate is less than 5%. Despite major progress in medical science during the last decades, the annual cancer-related death rate for PDAC has not changed significantly during these decades (Jemal *et al*, 2005). Additionally, therapies proven to be successful for treating many other tumour entities, failed in PDAC treatment.

Deregulated genes in PDAC have been published recently by us and others (Crnogorac-Jurcevic *et al*, 2002; Iacobuzio-Donahue *et al*, 2003; Logsdon *et al*, 2003; Grutzmann *et al*, 2004). These genes included candidates involved in cell cycle control, apoptosis, cell motility or other tumour relevant pathways, but druggable key players have to be defined yet. Thus, new therapies for PDAC are urgently required. Because given gene therapy, directly addressing single deregulated genes, still suffers from inefficient gene transfer and tumour targeting, we focused on other drugs like histone deacetylases (HDAC) inhibitors, generally influencing the activity of deregulated genes by changing the chromatin status. Acetylation of histones is associated with gene activation, whereas deacetylation mediated by HDAC is associated with gene silencing, classifying HDAC as a powerful drug target (Yoshida *et al*, 2001). Inhibitors of HDAC modulate chromatin structure resulting in loosening of the chromatin and changing transcription factor loading to the DNA (Nakano *et al*, 1997).

This modulates the expression pattern of various tumour relevant genes for the control of the cell cycle or apoptosis (Huang *et al*, 2000; Kim *et al*, 2004) causing inhibition of cell growth and differentiation (Feinman *et al*, 2002; Svechnikova *et al*, 2003; Yokota *et al*, 2004).

4-phenylbutyrate (4-PB) is one of few histone deacetylase inhibitors (HDACi) already tested in clinical trials in the treatment of recurrent malignant gliomas or the myelodysplastic syndrome (Bhalla and List, 2004; Phuphanich *et al*, 2005). Additionally, it is a FDA-approved and well-tolerated drug for urea cycle disorders (Rubenstein and Zeitlin, 1998), therefore being an attractive candidate for other therapies.

Gemcitabine is a widely used drug in the treatment of several cancer entities, including lung, breast and pancreatic carcinoma. Gemcitabine belongs to the antimetabolites interfering with the cell cycle progression. In several studies gemcitabine was combined with other established anticancer drugs like 5-FU, cisplatin, docetaxel and radiotherapy to address or to overcome the extraordinary resistance of pancreatic carcinoma to chemotherapy (Eisenberg *et al*, 2005; Ko and Tempero, 2005; Pipas *et al*, 2005).

In this study, we investigated the effect of 4-PB as a single drug on various cancer-related parameters like proliferation, induction of apoptosis and intercellular communication in several PDAC cell lines. Furthermore, although comparing the effects of single drug treatment with gemcitabine and 4-PB, we show overadditive effects in a combined treatment with both drugs. This study provides a comprehensive insight into the effects and advantages of the use of the HDACi 4-PB in the treatment of PDAC. Additionally, for the first time, we could show synergistic effects when combining 4-PB treatment with gemcitabine, a classical chemotherapeutical drug.

## MATERIALS AND METHODS

### Cell culture

Pancreatic carcinoma cell lines COLO 357, Panc 1, T3M-4; BxPc3 (Sipos *et al*, 2003) and primary human fibroblasts were grown as described (Grutzmann *et al*, 2004). HPDE6-E6E7-c7 cells (H6c7) (Furukawa *et al*, 1996; Ouyang *et al*, 2000) were grown in a medium containing FCS (10%, PAN Biotech, Aidenbach, Germany), RPMI 1640 (45%), Keratinocyte-SFM (45%), Bovine Pituitary Extract (25 mg l^−1^) and hEGF (2.5 *μ*g l^−1^, all Invitrogen, Karlsruhe, Germany). Cells were treated with 4-PB (Triple Crown America, dissolved in phosphate-buffered saline (PBS)), JNK inhibitor II (SP600125; Calbiochem, Merck, Darmstadt, Germany) or zVAD (Bachem, Weit am Rhein, Germany) as indicated. Cell counting was performed using a CASY1 TT-cell counter (Schärfe System, Reutlingen, Germany) according to the manufacturer's instruction.

### Isolation of human PBMC

Peripheral blood mononuclear cells (PBMC) were isolated by Ficoll–Hypaque density gradient centrifugation of buffy coat preparations obtained from healthy blood donors. T-cell blasts were generated from unseparated PBMC in the presence of phytohemagglutinin (PHA 0.5 *μ*g ml^−1^, Murex Biotech Ltd, Abbott, Wiesbaden, Germany). After 3 days, dead cells were removed by Ficoll density gradient centrifugation and T-cell blasts were expanded in RPMI 1640 with 5% FBS, antibiotics, L-glutamine and HEPES supplemented with 50 U ml^−1^ rIL-2 (Chiron Behring, Novurtis, Marburg, Germany). Cells were kept at 37°C in a humidified atmosphere with 5% CO_2_. For cytotoxicity/viability assays, freshly isolated PBMC or PHA blasts (day 14) were incubated for 20 h with or without gemcitabine (1.0 *μ*g ml^−1^) in the presence or absence of different concentrations of 4-PB from 0.5 to 5.0 mM. FACS analysis was performed as described below.

### Cell cycle analysis and accompanied apoptosis measurement

After two washes with PBS, cells were trypsinised, pelleted, resuspended in PBS containing 5 mM EDTA and fixed by adding one volume of ethanol (Merck). After RNase-treatment (40 ng RNaseA *μ*l^−1^, Sigma-Aldrich, Munich, Germany) cells were pelleted, resuspended in PBS containing propidium iodide (200 *μ*g ml^−1^) and subjected to FACS analysis. Cell cytometry was conducted using a FACScan cell analyzer (Becton-Dickinson Bioscience, Heidelberg, Germany). WinMDI2.8 (http://facs.scripps.edu) was used for analysing FACS data.

### Gap junction communication

Subconfluent cells were trypsinised, pelleted, resuspended in RPMI 1640 medium and divided into equal portions. Two samples were treated either without or with 1.0 mM or 5.0 mM 4-PB, respectively. Additionally, one of the corresponding cell portions was treated with calcein-AM (Molecular Probes, Invitrogen, Karlsruhe, Germany) at a concentration of 67 nM. After 40 min at 37°C, cells were washed three times in PBS and the corresponding portions treated with identical 4-PB concentrations were combined and plated onto a cell culture vessel. After 5 h additional incubation at 37°C, cells were trypsinised, washed, resuspended in PBS and analysed by FACS analysis.

Samples containing only unstained or stained cells served as a control and were also used for validating staining of the cells.

### Multi drug resistance

Cells were treated without or with 2.0, 5.0, 10.0 mM 4-PB or 100 *μ*M verapamil for up to 72 h. Then, Calcein-AM was added to a final concentration of 100 nM to the medium. After 2 h, cells were trypsinised, washed two times and resuspended in PBS. The intracellular accumulation of the dye was determined by cell cytometry (Karaszi *et al*, 2001).

### Apoptosis assays

The JAM assay was performed as described previously (Ungefroren *et al*, 1998). In some cases, apoptosis was also measured using ApoAlert annexin-V binding assay (Clontech, Saint-Germain-en-Laye, France) according to the manufacturer's protocol.

### Histone deacetylase activity assay

BxPc3 and T3M-4 cells untreated or treated with 2.0 mM 4-phenylbutyrate for 48 h were released by trypsinisation and washed in PBS. After centrifugation, the cell pellet was resuspendet in PBS and cells were lysed by three cycles of freezing and thawing followed by sonification. The HDAC activity in the supernatant was determined using the Histone Deacetylase Activity Assay Kit, colorimetric (Calbiochem) according to the manufacturer's instruction.

### Western blot analysis

Western blot analysis was performed as described before (Trauzold *et al*, 2003). Primary antibodies used were purchased from different companies as indicated: anti-PARP-mab (Ab-2, Calbiochem), anti-Caspase 8 (StressGen Biotechnology, Biomol, Hamburg, Germany), anti-Bid (R&D Systems, Wiesbaden-Nordenstadt, Germany), anti-*β*-actin (Sigma-Aldrich), anti-phospho JNK and anti-JNK (Cell Signaling, Danvers, MA, USA).

### Computer based analysis

*T*-test; ANOVA analysis and graphical presentations were performed using GraphPad Prism version 4.02. for Windows (GraphPad Software). Photoshop CS (Adobe) was used for preparing microscopic photos.

## RESULTS

### 4-phenylbutyrate inhibits HDAC activity in pancreatic cancer cells

**Figure 1:**
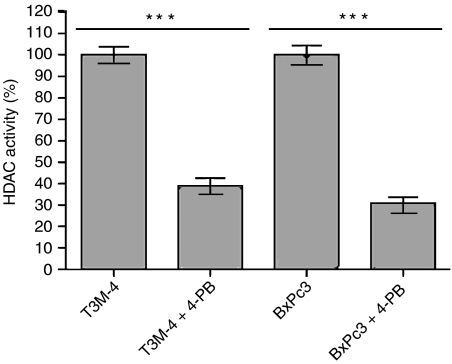
4-phenylbutyrate inhibits HDAC activity in pancreatic cancer cell lines. T3M-4 and BxPc3 cells were untreated or treated with 2.0 mM 4-PB for 48 h. Histone deacetylases activity of untreated cells was determined colorimetrically and set to 100%. The relative HDAC activity in treated cells was calculated. Results from five measurements are presented (mean±s.e.m). The observed inhibition proved to be highly significant (*t*-test; *P*<0.0001).

To verify the assumed inhibitory effect of 4-PB on the HDAC activity in pancreatic cancer cells, we determined the HDAC activity in T3M-4 and BxPc3 cells treated with the drug and compared the results with an untreated control. After 48 h of treatment, even concentration of 4-PB as low as 2.0 mM led to a 60–70% decrease in HDAC activity in both cell lines (Figure 1). Thus, 4-PB has a tremendous effect on the activity of the HDAC activity in pancreatic cancer cells.

### 4-phenylbutyrate treatment inhibits growth and induces apoptosis of ductal pancreatic adenocarcinoma cells

**Figure 2:**
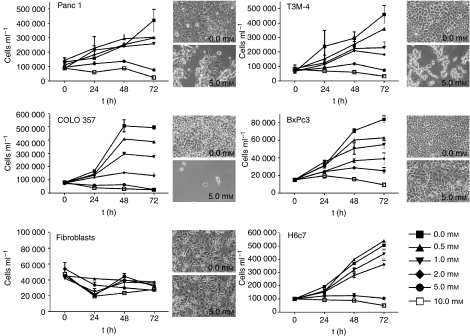
4-phenylbutyrate reduced cell number of pancreatic carcinoma cell lines *in vitro.* Panc 1, T3M-4, COLO 357, BxPc3, primary human fibroblasts or H6c7 cells were cultivated in the absence (black square) or presence of increasing 4-PB concentrations (0.5 mM (triangle), 1.0 mM (inverted triangle), 2.0 mM (diamond), 5.0 mM (circle)) up to 10.0 mM (white square). After 0, 24, 48 and 72 h, the cell number per 1 ml medium was determined from every sample by cell counting using the CASY1 TT-cell counter. Mean and s.e.m from at least three experiments are presented. To document morphological changes, a photo from each cell line taken after 48 h of treatment with 5.0 mM 4-PB (lower photo) and a corresponding untreated control (upper photo) is presented, respectively.

To study the influence of 4-PB on PDAC cells, we analysed growth, morphology and viability of T3M-4, BxPc3, COLO 357 and Panc 1 cells treated with different concentrations of 4-PB for up to 72 h. By counting cell numbers, we found that 4-PB significantly reduced cell growth in a dose-, time- and cell line-dependent manner (Figure 2). Whereas even low concentrations of 4-PB (1.0 mM) strongly inhibited growth of T3M-4, BxPc3, Panc 1 and COLO 357 cells, primary cultures of human diploid fibroblasts that grew very slowly appeared much less sensitive to 4-PB treatment. Concentrations up to 10.0 mM did not essentially influence number of these cells even after 72 h of treatment (Figure 2). Performing an ANOVA test, the correlation of the decrease in cell number and the concentration of 4-PB was found to be extremely significant for all pancreatic tumour cell lines (*P*<0.0001). Microscopical analyses revealed clear changes in the morphology of all pancreatic tumour cells tested (Figure 2), indicating cell death already 48 h after treatment with 4-PB. In contrast to human fibroblasts that remained unaffected in this regard, human pancreatic ductal epithelial cell line H6c7, which is rapidly growing comparable to the cancer cell lines is sensitive to 4-PB treatment when the concentration of the drug exceeds 2.0 mM.

As 4-PB-mediated growth inhibition could reflect induction of cell death or cell cycle arrest, we performed propidium iodide staining of cells treated for 48 h with increasing concentrations of 4-PB and analysed the percentage of cells in sub-G1 phase of the cell cycle.

**Figure 3:**
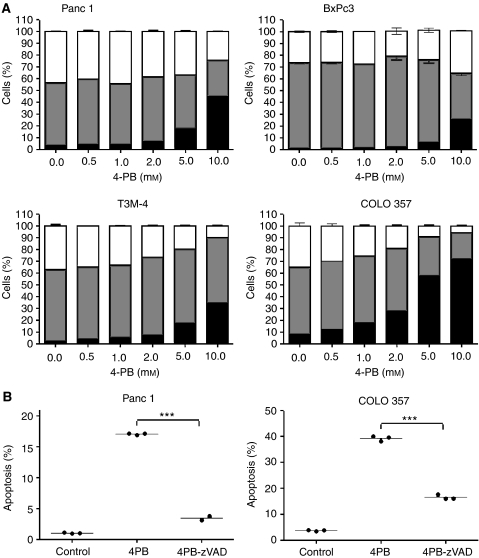
4-phenylbutyrate induced apoptosis and cell cycle arrest of pancreatic cell lines in a concentration-dependent manner. (**A**) Panc 1, BxPc3, T3M-4 and COLO 357 cells were treated with different concentrations of 4-PB for 48 h. Cells were trypsinised and stained with propidium iodide. The percentages of apoptotic cells (black), cells in G1-phase (grey) or S- and G2-phase (white) were determined by flow cytometry. The mean from at least three samples is presented. (**B**) ZVAD inhibited 4-PB induced apoptosis. Panc 1 and COLO 357 cells were treated either with 5.0 mM 4-PB alone or a combination of 5.0 mM 4-PB and 23 *μ*g ml^−1^ zVAD for 72 h. Cells were trypsinised and stained with propidium iodide. The percentage of apoptotic cells was determined by flow cytometry. Prism4 was used to perform *t*-test analysis. Effects of zVAD treatment proved to be highly significant (*P*<0.0001).

As shown in Figure 3A, 4-PB increased the number of cells in sub-G1 in all cell lines tested in a concentration-dependent manner. COLO 357 cells appeared most sensitive. Forty-eight hours exposition to 4-PB concentrations as low as 2.0 mM led to death of approximately 30% of these cells and more than 70% died when exposed to 10.0 mM 4-PB. Although Panc 1, BxPc3 and T3M-4 cells were more resistant to 4-PB treatment, higher 4-PB concentrations, for example, 10.0 mM also resulted in significant cell death. To prove mechanisms of cell death, we performed cell cytometry with Panc 1 cells and COLO 357 cells treated with 4-PB with or without pre-incubation with the broad spectrum caspase inhibitor zVAD-fmk. As shown in Figure 3B, cell death was significantly reduced in the presence of zVAD, indicating that the observed cell death was apoptotic. In parallel, cell cycle analysis showed a concentration-dependent cell cycle arrest of T3M-4 and COLO 357 cells. This arrest could not be demonstrated for Panc 1 and BxPc3 cells (Figure 3A), even after prolonged incubation times of 72 h. Thus, cell cycle arrest is no prerequisite for the induction of apoptosis. In contrast, normal mononuclear cells isolated from peripheral blood of human donors showed only weak response to treatment up to 5.0 mM 4-PB (Figure 5C and data not shown), underlining the tolerance of non-malignant cells for 4-PB.

### 4-phenylbutyrate increased intercellular communication between pancreatic carcinoma cells

**Figure 4:**
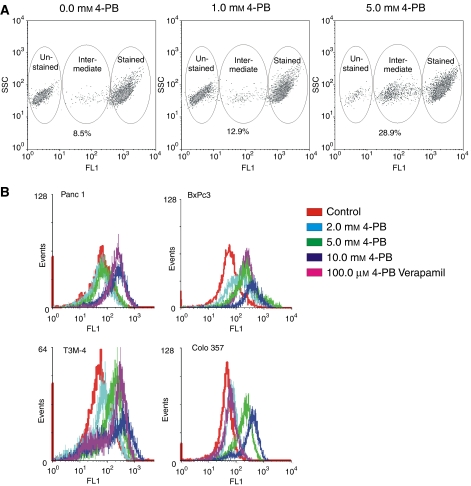
4-phenylbutyrate reduced cellular export and increased intercellular communication. (**A**) Pancreatic carcinoma cells either untreated, treated with 2.0 mM 4-PB, 5.0 mM 4-PB, 10.0 mM 4-PB or 100.0 *μ*M verapamil for 72 h were stained with Calcein-AM. The dye export was visualised by flow cytometry. The cellular export of calcein-AM was decreased by applying 4-PB in a dose-dependent manner (increase in fluorescence intensity) (**B**) Calcein-AM stained and unstained T3M-4 cells were co-cultured in the absence or presence of 4-PB (1,0 mM or 5,0 mM 4-PB as indicated) for 5 h. Cell cytometry was performed to distinguish unstained from calcein-AM stained cells. In the presence of 4-PB, the number of intermediately stained cells increased (right panel), whereas the number of unstained cells was reduced, showing intercellular dye transfer. The number of cells involved in dye transfer was calculated (% of ‘intermediately’ stained cells).

In order to investigate intercellular communications of adjacent tumour cells, T3M-4 cells were labelled with calcein. After trypsinising and washing, cells were plated onto unlabelled cells of the same origin. Calcein loaded cells can attach to and transfer the dye into non-labelled cells via gap junctions (Asklund *et al*, 2004). Because the concentration of the dye in these afore unstained cells (now ‘intermediately stained cells’, Figure 4A) is lower than in the pre-labelled cells, their fluorescence is also weaker. Thus, these cells can be identified by flow cytometry. When the tumour cells were cultured in the presence of 4-PB for 24 h preceding and during the dye transfer, the intercellular dye transfer was enhanced. Although 1.0 mM 4-PB induced dye transfer only marginally after 5 h of co-culture, it nearly tripled dye transfer at a concentration of 5.0 mM. Figure 4A shows a typical outcome of the experiment conducted three times.

### 4-phenylbutyrate inhibits cellular export mechanisms in a concentration-dependent manner

One reason for the poor response of PDAC to chemotherapy is the drug resistance achieved by membrane protein pumps (Glavinas *et al*, 2004) developed by this cancer. In order to investigate the effect of 4-PB on cellular efflux and to identify possible benefits of a combinatorial treatment with other chemotherapeutics, the ability of cancer cells to export calcein-AM added to the cell culture medium was determined by flow cytometry. If this ability of the cell is reduced, calcein-AM accumulates in the cell and becomes a substrate of cellular esterases. The product of this enzymatic reaction, calcein, is a strong fluorescent. Thus, the fluorescence intensity of a cell inversely correlates with the cellular export (Karaszi *et al*, 2001). Pancreatic carcinoma cell lines Panc 1, BxPc3, T3M-4 and COLO 357 were treated with 0.0, 2.0, 5.0 or 10.0 mM 4-PB or 100 *μ*M verapamil, a known inhibitor of MDR1-mediated efflux (Hindenburg *et al*, 1987) as a control to assure an adequate experimental design. Although Panc 1 cells only showed a response to 10.0 mM 4-PB after 72 h of treatment and this maximum response was comparable to verapamil treatment, BxPc3, T3M-4 and COLO 357 responded already to lower doses of 4-PB, COLO 357 even much stronger than to verapamil (Figure 4B). Interestingly, the effect of the drug on cellular export increases even more with the time of treatment (data not shown). These results demonstrated that cellular export mechanisms of pancreatic carcinoma cell lines can be effectively influenced by 4-PB.

### 4-phenylbutyrate increases gemcitabine-mediated apoptosis in pancreatic tumour cells

**Figure 5:**
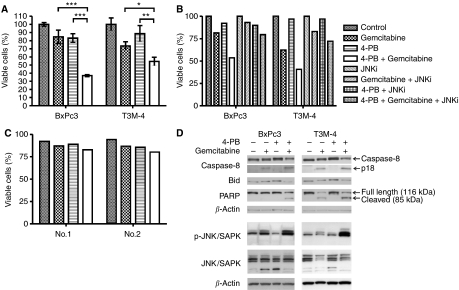
4-phenylbutyrate and gemcitabine induced apoptosis synergistically due to activation of Caspase 8 and BID in a JNK-pathway-dependent manner. (**A**) Cells were either not treated (fine dotted), treated with gemcitabine (1.0 *μ*g ml^−1^, dotted), 4-PB (1.0 mM, dashed) or gemcitabine and 4-PB simultaneously for 48 h and utilised for JAM assay. Mean and s.d of the number of viable cells (compared to control) from at least three experiments are shown. Differences between the single drug and the combinatorial treatment proved to be significant (*t*-test). (**B**) BxPc3 or T3M-4 cells either not treated or treated with gemcitabine (1.0 *μ*g ml^−1^), 4-PB (1.0 mM), JNKi (SP600125, inhibitor of c-Jun N-terminale kinase, 20 *μ*M) or different combination of these drugs for 48 h as indicated in the legend were stained by annexin V/propidium iodide and employed for cell cytometry. Number of viable cells is presented. The gemcitabine-induced cell death (as single drug or in combination with 4-PB) could be inhibited by inhibition of JNK. (**C**) Peripheral blood mononuclear cells were isolated from two human donors (no. 1 and no. 2) as described in Materials and Methods. Cells were not treated or treated with gemcitabine, 4-PB or a combination of both for 20 h. Cells were collected, stained by annexin V/propidium iodide and FACS analysis was conducted. The number of viable cells is presented. (**D**) After isolating proteins from BcPc3 or T3M-4 cells not treated (−) or treated (+) with 1.0 mM 4-PB and/or 1.0 *μ*g ml^−1^ gemcitabine for 48 h, 20 *μ*g protein were used to conduct Western blot analysis using specific antibodies against Caspase 8, Bid, PARP, phosphorylated JNK/SAPK, whole JNK/SAPK or *β*-actin as a control (p18: cleaving product of Caspase 8 activation).

Gemcitabine is one of the currently used substances to treat pancreatic adenocarcinoma. However, it does not improve the long-term survival of the patients (Bengala *et al*, 2005; Gelibter *et al*, 2005). Here, we tested the influence of concomitant use of 4-PB and gemcitabine on two resistant pancreatic adenocarcinoma cell lines T3M-4 and BxPc3. Using DNA-fragmentation assay (JAM, Figure 5A) as well as annexin V/propidium iodide staining (Figure 5B), we found that 4-PB strongly enhanced gemcitabine-induced apoptosis in both cell lines. As shown in Figure 5A, low dose of gemcitabine (1.0 *μ*g ml^−1^) resulted in BxPC3 cells in only moderate cell death. Similarly, low dose of 4-PB (1.0 mM) only slightly induced cell death. Interestingly, concomitant treatment of cells with both, gemcitabine and 4-PB strongly enhanced the effects of each of the substances alone and over 60% (determined by JAM assay, Figure 5A) or 50% (FACS analysis, Figure 5A) of cells died after 48 h of treatment. Similar results were obtained with T3M-4 cells (Figure 5A and B). Higher doses of 4-PB (2.0 mM and 5.0 mM) only slightly increased the sensitising effects (data not shown).

In contrast to PDAC cell lines, PBMC isolated from human donors and cultured *in vitro* were much less sensitive to the drug treatment and did not show a significant response to the combinatorial treatment compared to the single drug treatment (Figure 5C).

Western blot analyses (Figure 5D) of lysates prepared from cells treated with 4-PB, gemcitabine or with both agents demonstrate that 4-PB strongly enhanced gemcitabine-mediated Caspase 8- and Bid-cleavage as well as PARP-cleavage assayed as a marker for Caspase 3-activation (Figure 5D).

Interestingly, 4-PB treatment led in both cell lines to upregulation of the expression of Caspase 8 and Bid. No differences in the expression levels of Caspase 3, Caspase 2 and IAPs were found in 4-PB-treated cells compared to untreated controls.

Recently, it has been shown that in gemcitabine-sensitive pancreatic tumour cell lines PK-1 and PCI-43, the drug-mediated apoptosis required the activation of p38 mitogen-activated protein kinase (MAPK) (Habiro *et al*, 2004). Thus, we analysed the involvement of p38 as well as MEK and JNK in gemcitabine-mediated apoptosis of BxPc3- and T3M-4 cells that were only moderately sensitive to gemcitabine treatment. Using specific MAPK inhibitors, we found no influence of MEK or p38 on gemcitabine-mediated cell death in these cells (data not shown). In contrast, inhibition of JNK completely abolished the sensitising effect of 4-PB (Figure 5B). Western blot analyses revealed that treatment of both cell lines with gemcitabine led to an increased JNK activity, the effect that was strongly enhanced by 4-PB (Figure 5D).

## DISCUSSION

In several current gene expression studies of pancreatic tumour cells, the authors have identified numerous genes differently expressed in tumour tissue compared to normal tissue (Crnogorac-Jurcevic *et al*, 2002; Iacobuzio-Donahue *et al*, 2003; Logsdon *et al*, 2003; Grutzmann *et al*, 2004) in order to identify possible therapeutic targets. But no key player whose expression could be modified in the context of a specific therapy has been identified yet.

Instead of addressing the expression of only one or few specific genes, drugs influencing general mechanisms of gene regulation could address the aberrant expression of various genes simultaneously (Sowa and Sakai, 2000). As low differentiation grades of carcinomas correlate with a bad prognosis, drugs inducing a differentiated phenotype could be a substantial benefit for patients with tumours like PDAC. Either single drug treatment itself, or more likely, combinatorial treatment with established anticancer regimens, might prove to be efficient by downregulating genes responsible for apoptosis resistance. Acetylation of histones, for example, by transcription factors induces changes in the chromatin structure and increases the accessibility of the DNA leading to gene activation. On the other hand, deacetylation of histones by HDAC inactivates gene expression (Kuo and Allis, 1998; Hess-Stumpp, 2005). Inhibitors of HDACs might be a promising additive in cancer therapy as they can activate numerous genes and thus might reactivate pathways controlling apoptosis, differentiation or growth control (Huang *et al*, 2000; Sowa and Sakai, 2000). The HDAC inhibitor 4-PB (Jung, 2001) belongs to the family of short fatty acids and is long used for treatment of inborn defects of the urea cycle. Patient suffering from this disease have been treated with 4-PB for years without major side effects at a similar dose that was used in this study (recommended treatment for urea cycle disorders: 3.0 g kg^−1^ bodyweight day^−1^ according to the manufacturer; http://www.tributyrate.com/html/urea_cycle_disorder.php).

Here, we demonstrated that 4-PB efficiently increases gap junction communications (GJCs) and inhibits cellular export mechanisms in PDAC cell lines along with overcoming drug resistance, induction of apoptosis and reduction of tumour cell numbers.

An induction of apoptosis upon 4-PB treatment is in agreement with studies showing this in cancer cell lines derived from other tumour entities like prostate cancer (Carducci *et al*, 1996), medulloblastoma (Li *et al*, 2004) or colon cancer (Feinman *et al*, 2002). Furthermore, in a rat model system 4-PB caused regression of tumours derived from hepatocarcinoma (Hep3B) or hepatoblastoma (HepT1) cells (Svechnikova *et al*, 2003).

Interestingly, we found that H6c7- cells, a pancreatic ductal epithelial cell line is also sensitive to 4-PB treatment with concentrations exceeding 2.0 mM. Even if not from malignant origin and with nearly normal phenotype, these E6/E7 transformed cells (Furukawa *et al*, 1996; Ouyang *et al*, 2000) bear some characteristics of tumour cells like immortality and a high proliferation rate. Thus, our data support the idea that 4-PB acts not specifically on malignant but also on other highly proliferating immortalised cells. Low proliferating primary human fibroblasts and PBMC were not effected by 4-PB in the concentrations tested. Partly in contrast to other studies reporting a cell cycle arrest of tumour cells upon 4-PB treatment (DiGiuseppe *et al*, 1999; Clarke *et al*, 2001), only half of the cell lines investigated by us showed an arrest of the cell cycle in the G1-phase. This divergence namely of the PDAC cells might be explained by significant differences in the transcriptome of these cell lines (Grutzmann *et al*, 2004). It has been previously described that the cell cycle arrest in tumour cells upon 4-PB treatment depends on p21 (DiGiuseppe *et al*, 1999). Interestingly, according to a microarray-based study (Grutzmann *et al*, 2004), cells showing a cell cycle arrest in this study (T3M-4 and Colo357) express p21 to a higher extend than the non-arresting cells (Panc1 and BxPc3). Whether this is indeed the reason for the differential behaviour upon the drug treatment or if other differentially expressed cell cycle regulators play a pivotal role remains to be shown.

Gap junctions are important for the transfer of small molecules between adjacent cells. The resulting intercellular communication is essential for both proliferation and activation of differentiation pathways (Seo *et al*, 2006). It has been shown that highly proliferating malignant cells have less gap junctions than more differentiated ones (Yamasaki and Naus, 1996). Interestingly, we found that 4-PB increases GJC in PDAC similar to previous analyses in glioblastoma (Asklund *et al*, 2004). Besides the fact that 4-PB already induces apoptosis of pancreatic carcinoma cells when applied as a single drug, the increase in GJC might allow an exchange of apoptotic signals between neighbouring cells. Furthermore, an increase in GJC is of benefit when additional anticancer drugs are administrated simultaneously and especially when bystander killing effects are important (Ammerpohl *et al*, 2004).

Upon chemotherapy tumour cells often gain resistance to established drugs. One way to achieve this resistance is the activation of export mechanisms. Cellular pumps like ABCG2, MDR1 or MRP1 can export a broad spectrum of molecules, including chemotherapeutics out of the cells (Glavinas *et al*, 2004; Katragadda *et al*, 2005), preventing them from killing the tumour cell. In this study, we could show that 4-PB reduces the cellular export of calcein-AM in all cell lines tested. In the case of COLO 357 and BxPc3 cells, the inhibition by 4-PB is even stronger than the one caused by verapamil, a known inhibitor of MDR1 (Hindenburg *et al*, 1987). Because concentrations of verapamil up to 500 *μ*M could not further inhibit cellular export in our experiment (data not shown) it is likely, that the verapamil concentration applied led already to a maximum inhibition of MDR1. Thus, the additional inhibition by 4-PB compared to verapamil is probably caused by the inhibition of additional or other cellular pumps than MDR1, which are not effected by verapamil. In contrast to verapamil, which as a ‘classical’ inhibitor caused maximum inhibition in less than 2 h (data not shown), 4-PB needed at least 24 h to cause any inhibition of cellular export and furthermore this inhibition increased with the time of treatment. Because high activities of cellular transporters were found to be associated with low differentiation grades (e.g. like in stem cells) (Islam *et al*, 2005a, 2005b), the decrease in cellular export caused by 4-PB might be due to induction of differentiation. This would be supported by other reports describing the potential of 4-PB to induce differentiation in tumour cells (Samid *et al*, 1997; Bar-Ner *et al*, 1999; Li *et al*, 2004). Thus, our findings that 4-PB increases the susceptibility of pancreatic carcinoma cells to apoptosis, that it increases intercellular communications and reduces cellular export makes 4-PB a promising drug for a combinatorial treatment with a ‘classical’ chemotherapeutical drug like gemcitabine or 5-FU, because higher differentiations grades are also associated with increased sensitivity to chemotherapy.

As gemcitabine is an established drug in the regimen against pancreatic adenocarcinoma, we combined this drug with 4-PB and tested the responsiveness of two pancreatic tumour cell lines BxPC3 and T3M-4. Most promising for future therapies, we found that 4-PB clearly potentiated gemcitabine-mediated cell death. Analysing the 4-PB-mediated sensitising effect, we demonstrated that 4-PB increased the expression of proapoptotic proteins Caspase-8 and Bid and strongly enhanced the gemcitabine-mediated activation of JNK. Although in other model systems like colon cancer or liver cancer induction of Caspase 3 by 4-PB treatment was described (Clarke *et al*, 2001; Svechnikova *et al*, 2003), we found only Caspase 8 to be upregulated.

Recently, it has been reported that high constitutive activity of NF-*κ*B inhibits chemotherapeutic agent-mediated apoptosis in pancreatic tumour cells (Arlt *et al*, 2003). By using EMSA and NF-*κ*B ELISA, we found no influence of 4-PB on NF-*κ*B activity (data not shown). We conclude that 4-PB does not influence this antiapoptotic pathway. Instead, inhibition of JNK-activity by JNK inhibitor II not only partially reduced gemcitabine-mediated apoptosis but also completely abolished the sensitising effects of 4-PB. This finding is in line with a recent study demonstrating the relevance of the JNK-pathway for apoptosis induction upon single drug 4-PB treatment in lung carcinoma cells (Zhang *et al*, 2004). In our model system, inhibition of the JNK-pathway primarily decreased the gemcitabine depending cell killing.

In contrast, Habiro *et al* (2004) showed that JNKs are not activated following gemcitabine treatment in pancreatic tumour cell lines PK-1 and PCI-43 and are dispensable for gemcitabine-induced apoptosis and in these cells. Instead, the authors postulated the involvement of p38 MAPK in this apoptotic pathway, the effects which we could not confirm in BxPC3 and T3M-4 cells, even using the same pharmacological inhibitor SB203580 (data not shown). These discrepancies may be explained by the cell lines used for investigations. Nevertheless, our results demonstrate that gemcitabine induces JNK in BxPC3 and T3M-4 cells and this effect is partially involved in gemcitabine-mediated cell death observed in these cells. Moreover, 4-PB strongly increases JNK-activity and this obviously represents one of the mechanisms by which 4-PB sensitises cells towards gemcitabine therapy.

Interestingly, the sensitising effects of 4-PB were observed in low concentration of both 4-PB and gemcitabine and no significant further enhancement of apoptosis could be achieved neither by the increase of 4-PB concentration nor by the increase of gemcitabine (data not shown).

Highly relevant for the clinical use, 4-PB acts specifically on malignant cells, as non-malignant cells like primary human fibroblasts and PBMC were significantly less sensitive to this treatment. This important finding is supported by other authors showing the insensitivity of HBE4-E6/E7-cells, a non-transformed cell line, to 4-PB treatment (Zhang *et al*, 2004). This tolerance of non-malignant cells makes 4-PB an interesting drug also in a combinatorial therapy with established chemotherapeutics where side effects would prevent a combination with other cytotoxic drugs.

4-phenylbutyrate has been shown to have additional anticancer effects non-related to HDAC inhibition, for example, by inducing glutamine depletion (Darmaun *et al*, 1998). Even if we cannot rule out similar effects in pancreatic cancer cells, which could also contribute to the anticancer effect of the drug, a major contribution to the chemosensitising effect of 4-PB is very likely given by the inhibition of HDAC activity. The inhibition of HDAC activity probably induces changes in the expression of genes involved in cellular export mechanisms or GJC. This is also supported by findings in erythroleukemia cells and human leukaemic cells (Lea *et al*, 1999) denying a growth inhibitory effect depended on glutamine depletion upon 4-PB treatment. Further own findings by RT–PCR analysis showing an upregulation of tumour suppressor genes like fgfr2 or a downregulation of oncogenes like gadd45b, ccnb1, cflar or cdc25b in pancreatic cancer cells upon 4-PB treatment (data not shown) argue also for the involvement of general transcription regulating mechanisms like chromatin modifications, as these genes have been previously described to be deregulated in PDAC (Grutzmann *et al*, 2004).

Taken together, these findings provide evidence that the well-tolerated and FDA-approved drug 4-PB (Rubenstein and Zeitlin, 1998) is to be seen as a promising supplemental therapeutic agent for the treatment of PDAC patients.
